# Post-Traumatic Stress Disorder (PTSD) Symptoms in Secondary Stalked Children of Danish Stalking Survivors—A Pilot Study

**DOI:** 10.3390/ijerph16050725

**Published:** 2019-02-28

**Authors:** Ask Elklit, Lene Annie Gregers Vangsgaard, Anne Sophie Witt Olsen, Sara Al Ali

**Affiliations:** Department of Psychology, University of Southern Denmark, Campusvej 55, DK-5230 Odense M, Denmark; Lgv@live.dk (L.A.G.V.); annesophiewo@gmail.com (A.S.W.O.); salali@health.sdu.dk (S.A.A.)

**Keywords:** stalking survivors, secondary stalking, children, trauma reactions, PTSD

## Abstract

There is a lack of research examining secondary stalking and its effect on children who, in many cases, can be direct targets, or secondary survivors, of the stalking of their parent. The present study examines trauma reactions in children of stalking survivors in a Danish sample. It investigates the differences and similarities of such reactions across three age groups. Fifty-seven children were divided into groups depending on their age. The symptoms of the youngest group, 0–6-year-olds, were investigated by way of a maternal diagnostic interview. The two older groups, 7–11- and 12–19-year-olds completed the age-appropriate questionnaires, “Darryl” and “HTQ”, respectively, online. Twenty-two percent of the youngest group met the criteria for Post-Traumatic Stress Disorder (PTSD). Eighty-five percent of the middle age group and 58% of the older age group met PTSD diagnostic criteria. The findings illustrate that reactions to secondary stalking were predominantly within the arousal cluster of PTSD symptomology, with sleep disturbances and irritability commonly reported. The overall prevalence of children meeting PTSD diagnostic criteria in the sample was 56%. Future studies will benefit from larger samples and from knowledge of any pre-existing relationship between parent and stalker.

## 1. Introduction

Stalking may be defined as “a course of conduct directed at a specific person that involves repeated visual or physical proximity, non-consensual communication, or verbal, written or implied threats, or a combination thereof, that would cause a reasonable person fear” [1]. In Denmark, 9% of the adult population have been stalked during their lifetime [2,3], and each year, 100,000–132,000 Danes are exposed to stalking [2,4]. 

Studies investigating reactions to stalking demonstrate that survivors exhibit an increased risk of developing depression, anxiety, PTSD, and other mental disorders [5,6]. The Schandorph and Elklit [3] study found that 75% of mothers stalked by their ex-partner met the criteria for a PTSD diagnosis, with a further 17% meeting the criteria for subclinical PTSD. The study also reported that these survivors exhibited a predisposition to lifestyle complications by means of a diminished aptitude in dealing with occupational and psychological life factors. The mothers reported an intense feeling of powerlessness when witnessing their children’s reactions to stalking, with several mothers stating that their perceived parental abilities were adversely affected [3,7]. Furthermore, the study also demonstrated that around 95% of the survivors were either married, separated/divorced, or an ex-partner of the perpetrator [3] which suggests that children of survivors may be in close proximity in terms of witnessing the effects of the stalking. Such findings are of note, with statistics demonstrating that approximately 37% of Danish stalking survivors have children in the home [4].

Previous studies have for example investigated how PTSD, depression, and anxiety influence the relationship between mother and child [8]. A child’s development is greatly influenced by the physical and emotional protection provided by their caregiver in times of distress [8]. In instances where these protective factors are compromised, and where the children have witnessed aspects of the stalking behavior, such offspring are likely to be at greater risk of developing mental disorders [3,7]. 

It is important to be aware of children experiencing traumatizing events early in life, because of their developmental vulnerability and the high risk of trauma influencing their long-term life outcomes [7]. Cohen, Mannarino and Deblinger [9] reported that trauma in early childhood has a major influence on a child´s developmental trajectory. Such studies have demonstrated a significant association between childhood adversity and subsequent adult onset of psychiatric diagnoses [10], health risk behaviors [11], and diminished physical health [8,10,12]. 

Clinical observations and research has sought to explain that the consequences of trauma are not limited to the person who has experienced it, but also significant others in their environment, such as their children. This phenomenon has been called secondary traumatization [13]. The theory around secondary traumatization assumes that when a parent suffers from a trauma, the trauma can also affect their child’s function and development. The parents can carry over their psychological symptoms to their children, either directly, by the child developing the same symptom, or indirectly, by the child getting affected by his/her parents’ traumatization, and therefore develop psychological problems [14]. In this case, the children can therefore also be viewed as secondary survivors of the traumatic stalking experienced by their mother. The Schandorph & Elklit [3] study shows that the perpetrators threats and behavior affect the mothers to the extent where many of them feel unsafe in their own homes, and even feel like they have lost control over their own lives. One could therefore argue that because of this, the children in this case could be secondary traumatized by their mother’s traumatized condition. Therefore, it is important to examine whether PTSD could be a possible outcome in the children of survivors, since such a high percentage of the mothers who were stalked met the criteria of a PTSD diagnosis [3].

Furthermore, the A criterion in the DSM-V PTSD diagnosis [15] explains that you could be traumatized by an event by direct exposure to it, witnessing it, learning that a relative was exposed to it or indirectly by exposure to distressing details of an event, such as repeatedly hearing details about it (DSM-V). Children of stalked parents could certainly be assumed to have experienced the traumatic event in one or more of these ways, either by directly being a part of it or witnessing it as it happens to their parent, or by learning or hearing about it.

Despite a growing interest in stalking and its psychological sequelae, there is a relative paucity of research examining the effect of stalking on children who in many cases are direct targets, or secondary survivors of the stalking behavior [6,15]. Secondary traumatization is also a relatively new area of research, despite the growing literature [16]. The present study examines trauma reactions in children of stalking survivors in a Danish sample. It investigates the differences and similarities of such reactions across three age groups. It is relevant to examine whether children in different developmental stages show different reactions, since there are certain differences in the way they show PTSD symptoms in general, which is explained later in the section about the age groups. The distinction between the three age groups in the current study are equivalent to preschoolers (0–6 years), primary school children (7–11 years), and secondary + high school children (12–18 years).

### 1.1. PTSD

Stalking is often perceived as life-threatening by the person being stalked, due to fear of being the target of personal violence [6]. The Schandorph & Elklit [3] study showed that around 99% of the stalked women had experienced psychological violence, and 96% had experienced physical violence from their stalker. The threat and anticipation of implicit and explicit violence, mentally or physically, establishes stalking as a behavior comparable to domestic violence. Some traumas occur as a single event trauma, which is sudden and unexpected, while other traumas occur as a result of multiple events, or a series of events through a period of time [17]. Stalking may be considered a multiple-event continuous trauma, also called a complex trauma [18], and a child residing with a survivor of stalking is living with a person in constant fear and arousal [6]. Such caregivers, who themselves are traumatized, may not have the capacity to detect their child’s distress [8] 

Diagnosing PTSD in children and adolescents who have experienced multiple traumas can be difficult due to diminished communication skills, an unwillingness to convey their experiences, and fear of disclosure [7,8,19]. The diagnostic criteria for PTSD in children and adolescents are quite similar to that of adults, with diagnosis centering on three symptom clusters: re-experiencing (cluster B), avoidance (cluster C), and arousal (cluster D) [11,20,21]. The symptoms are experienced differently depending on age and developmental stage [21]. 

#### 1.1.1. Preschoolers (Age 0–6)

Studies demonstrate that preschoolers show symptoms of re-experiencing via post-traumatic play, for example, re-enacting themes of the trauma [11,22] and also via flashbacks, nightmares, or intense emotional or physical reactions to reminders of the event. Children may experience dissociative episodes, in which the child freezes and becomes unresponsive [11], and avoidance symptoms that may be subtle or pronounced, and can be observed in withdrawal from social interactions and in regression and denial [8], shown in limited engagement in conversations detailing reminders of the trauma. Arousal is often observed by sleep disturbances, increased irritability, temper tantrums, increased fussiness, hypervigilance, poor concentration, and increased activity levels [11].

#### 1.1.2. Schoolchildren (Age 7–11)

Schoolchildren often re-experience details via intrusive sounds or images. They can be observed inventing trauma-related games and experiencing symptoms such as trauma-specific fears, the development of new fears, increased reactivity, somatic complaints, nightmares, and recurrent revenge or rescue fantasies [11]. Avoidance symptoms can be occurrent by phobic behavior, truancy, sadness, guilt, loneliness, withdrawal from peers and play, and a feeling of having a limited future. According to De Young et al. [8], and Kerig et al. [11], dissociative episodes are similar to those experienced by preschoolers. Increased arousal is observed via a range of symptoms, such as difficulties falling asleep, oppositional defiance, diminished academic aptitude, obsession with trauma details, and an exaggerated startle response [11]. The traumatic experience can influence their capacity to express affection [23] and also increase risk behavior beyond their age-appropriate capabilities [23]. 

#### 1.1.3. Adolescents (Age 12–18)

Analogous studies indicate that adolescents report re-experiencing of traumatic events via recurrent revenge and rescue fantasies, nightmares, flashbacks, trauma-specific and mundane fears, and somatic pain. Arousal symptoms such as insomnia, withdrawal into heavy sleep, anger, aggression, and social withdrawal are commonly disclosed [11]. Their inability to cope with routine and daily life may cause them to isolate and withdraw from social interaction or act out [11,24]. They also demonstrate alterations in arousal and mood by the higher risk of engaging in risky or destructive behaviors, e.g., substance abuse, eating disorders, truancy, and violence [11,24,25]. 

Secondary stalking and its effect on children is an area of research that is unexplored. This novel study will therefore investigate trauma symptoms in children as a reaction to stalking of a parent, and secondly, compare the reactions across three age groups. Based on the evidence presented in the prior paragraphs, we expect that a high rate of children across the three age groups will suffer from PTSD, or at least experience high levels of several PTSD symptoms. Furthermore, it could be expected that the level of PTSD symptoms would be lowest in the youngest group because of their cognitive abilities compared to the older children.

## 2. Materials and Methods

### 2.1. Participants

Participants were parents recruited via the organization, “Danish Stalking Center”, and related groups on social media, and their children. The total number of participants in this study was *N* = 57; 18 parents who were interviewed about their preschool children in the age of 0–6 years, and 39 children between 7 and 19 years old. The mothers who had been stalked and had children between 0 and 19 years old, who responded to the recruiting, were all included in the current study. The only excluded mothers were the ones whose children were over the age of 19. The inclusion criteria of the children were if they were between 0 and 19 years and had mothers who were stalking survivors.

### 2.2. Procedure

The participants were divided into three groups. The participants in the age group 0–6 years (median age: 4) were assessed through a telephone interview with the mother, using the semi-structured interview guide “Diagnostic Infant and Preschool Assessment (DIPA)”. The interviews were conducted by a trained assistant and the mothers received a small compensation for their participation. Eighteen DIPA interviews were completed.

The participants in the second group (7–11-year-olds, median age: 9) were asked to complete an online version of the cartoon-based “Darryl test” on Survey Exact. A link to the test was sent to the mother by email, social media (Facebook), or a paper copy of the test was given to the mother to give to her child. Twenty children completed the test.

The third group of participants (12–19-year-olds), were asked to complete the Harvard Trauma Questionnaire (HTQ) on Survey Exact. The link was given directly to them or to their mother. Nineteen participants completed the assessment.

Informed consent was collected from the caregivers of the preschool children, and verbal assent was collected from the two older groups. Ethical guidelines from the Ethical Principles of Nordic Psychologists were adhered to, and data collection procedures were approved by the Danish Data Protection Agency.

### 2.3. Tests

The Diagnostic Infant and Preschool Assessment (DIPA) is a clinical semi-structured interview that is administered to the caregiver [26]. DIPA has been systematically validated and shown to be a sensitive tool when measuring psychiatric disorders in preschool children [26,27]. The test consists of 517 questions used to identify symptoms across 13 different diagnoses.

DIPA is divided into diagnostic modules that each begin with a fixed question. To enhance the diagnostic accuracy, the interviewer can, if necessary, ask clarifying questions if needed for a specific diagnosis. The modules consist of several categorical questions examining psychiatric symptoms based on the child’s observable behavior.

If the child has symptoms that negatively influence his/ her daily functioning, the caregiver may be asked about his/her own adjustments that may accommodate this diminished level of functioning at a familial level. The adjustments made by caregivers are measured on a 4-point Likert Scale ranging from “always” to “never”. By including the Likert scale on caregiver adjustment, the examination becomes more detailed and valid with regard to the daily functioning of the child. DIPA therefore provides a thorough, detailed description of symptoms, how they impair normal functioning, and how the caregiver subsequently adjusts his/ her behaviors.

The validated Danish version of DIPA was used in the present study [28]. It has shown good internal consistency for the overall scale of PTSD (α = 0.82), which was the only module used in this study. The diagnostic symptoms are based on the Diagnostic and Statistical Manual of Mental Disorder 4th edition, the revised version [21].

The cartoon test “Darryl” is a screening tool which is used to identify and measure PTSD symptoms in children and adolescents [29]. The screening tool has demonstrated good internal consistency for PTSD symptomology as detailed in the DSM-IV. Studies have shown that Darryl assesses PTSD symptoms in a developmentally appropriate manner [30]. The test is designed to be used on children between the ages of 5 and 18. The employed version consists of 23 cartoons of a pre-adolescent boy named Darryl, and three thermometers showing “never” (the thermometer is empty), “some of the time” (the thermometer is half full), and “a lot of the time” (the thermometer is full). The interviewer reads a short text matching the feelings of Darryl illustrated in the cartoon. The interviewer then asks the child to circle the thermometer that illustrates how often the child feels the same. The text alongside each cartoon asks about the psychological responses specific to the traumatic experience, in this case, stalking. In a validation study of the Danish version of Darryl, it showed good internal consistency for the overall scale (α = 0.88) [31].

The Harvard Trauma Questionnaire (HTQ) is an assessment tool in the form of a self-reporting questionnaire used in different trauma related assessments on adolescents and adults [32,33]. This study uses HTQ to measure trauma reactions in adolescents. The test contains 17 questions examining DSM PTSD symptoms [15]. The items are scored on a 4-point Likert scale, with 1 being “not at all” and 4 being “very often” [34]. A revised version of the HTQ was used in the present study, in which the questionnaire wording was adapted to stalking [33]. The HTQ has been reported to have high reliability and validity, internal consistency, test–retest reliability, and concurrent validity [33,35,36].

Since many of these assessment tools have yet to be systematically validated for the DSM-5, the DSM-IV is referenced in the current study.

### 2.4. Analysis

Results were analyzed using SPSS Version 24.0 (SPSS Inc. Chicago, IL, USA). A PTSD symptom algorithm, based on DSM-IV, was used to determine how the children were affected by stalking, and how often they experienced symptoms within the last month. This algorithm requires one (or more) symptoms from the re-experiencing cluster, three (or more) symptoms from the avoidance cluster and two (or more symptoms) from the arousal cluster. The results are summarized in Table 1.

## 3. Results

The results from the DIPA test reported that 22% of preschool children met the criteria for PTSD. In the Darryl test, 85% of participants met the PTSD criteria. Then, 58% of the adolescents met criteria for PTSD, as measured by the HTQ. The overall prevalence of children meeting PTSD criteria in the sample was 56%.

Table 1 shows Cluster B, C, and D PTSD symptoms presence in pre-schoolers, in schoolchildren “some of the time” or “a lot of the time”, and in adolescents “often” or “very often”, respectively, within the last month. The table also shows the presence of the aforementioned symptoms interference as a problem in the children’s daily life.

## 4. Discussion

The results show that children experience symptoms of trauma following exposure to stalking of their parent. The three groups differ in symptom prevalence, although they do show the same pattern over almost all symptoms. As hypothesized, the level of PTSD symptoms was the lowest for the youngest group. This is illustrated in the elevated number of schoolchildren and adolescents who meet the criteria for PTSD, when compared with their preschool counterparts.

This result differs from analogous studies in which preschoolers have been reported to be more susceptible to distress and developmental problems following traumatic exposure. This has been hypothesized to be resultant of their limited cognitive capabilities when faced with a traumatic event [8,9,37,38]. However, the limited cognitive abilities of preschoolers might also explain why the PTSD symptoms of this group is lower, since they may not conceive the consequences of potentially dangerous situations, and could, because of this, become less traumatized than the older children.

In the preschool group, symptoms were assessed via an interview with the stalked parent. This methodological application calls for two considerations: firstly, parents tend to be influenced by their own personal trauma, which may influence the way they assess their child’s well-being [25,37], and secondly, several PTSD symptoms are internalised, which means that it can be difficult to assess if they are experienced by a child. Young children may not have the cognitive abilities or language capacity to convey such symptoms. Moreover, children who are old enough to communicate may withhold feelings of anxiety or anger as a way of protecting their traumatised parent, or for fear of being caught between their parents [7].

Scheeringa and colleagues [39] addressed the issue of diagnosing PTSD in young children. They emphasize the fact that young children who suffer from PTSD may go untreated as they are unable to convey their symptoms due to diminished cognitive ability and low language competency. They put forward an alternative PTSD diagnostic tool where preschoolers are required to meet one symptom across clusters B, C, and D, and at least one symptom in a new symptom cluster, to achieve a diagnosis of PTSD. The new symptom cluster details psychological factors unrelated to trauma, i.e., new separation anxiety, new aggression, and new fears. By using this tool, preverbal children are taken into account, and diagnostic criteria expands to include behavioral observations.

Cluster D (“Arousal”) is the most common symptom cluster across the three groups, with the majority of participants reporting issues linked to sleep difficulties and nightmares. Furthermore, the majority of participants in the two older groups reported concentration difficulties. The high number of arousal symptoms may be related to behavior of the stalked parent, who is often on guard in a hypervigilant state, prepared for a new attack [16]. This could point to a possible direct secondary traumatization, where the child develops the same symptom as the traumatized parent.

Prevalence distribution of symptomology, alongside the number of participants meeting PTSD criteria, suggests that schoolchildren experience the highest level of psychological distress. The prevalence of PTSD in the total sample was 56%. Based on this finding, it is assumed that stalking of a child’s parent, and a child´s awareness of the stalking, could probably be associated with a myriad of psychological sequela, which can further be explained by the theory of secondary traumatization.

This emphasizes the importance of creating a supportive environment for a child whose parent is being stalked. Furthermore, it underlines the need for trauma treatment for the stalked survivors in order to prevent the indirect transmission of trauma to the child, where the child can be shown to be heavily affected by the parent’s trauma.

## 5. Limitations

The current study is a pilot study, and certain limitations must be considered in the present study. First and foremost, the findings of the study points at possible secondary traumatization of the children of stalking survivors. However, whether this traumatization is direct or indirect cannot be established, as PTSD symptoms in the parent was not assessed in the current study. Secondly, the combining of three different age groups, yet using different measures of PTSD across the groups that seem to be comparable only to a certain extent. This makes it difficult to determine whether the differences in prevalence are due to the age differences or whether they could be a result of the differences in measurement tools. DIPA and Darryl are both based on measuring PTSD according to the DSM-IV, and future studies could benefit from measuring DSM-5 PTSD across the different age groups. Overall, this requires further research to examine the analysis according to the DSM-5. It is also unclear whether the PTSD symptoms as measured by DIPA are linked to stalking victimization, or whether they could be a result of other traumatic events, as DIPA did not measure PTSD symptoms specific to the stalking event. Furthermore, the small sample size could also be a limitation, especially when the 57 subjects are divided across three age groups.

Stalking survivors hide to avoid their stalker, and experience high levels of anxiety about the possible exploitation of identity. This makes the stalked mothers very hard to find, as is also the case in the present study, where they have been found in a closed and hidden network. Therefore, much information and demographics about the stalked population is lacking, for example information about each woman’s degree of exposure to stalking. The women’s degree of exposure has though been described on a group level in another study [40]. Furthermore, this makes their children even harder to establish contact with, and more information about the children is therefore also lacking. It is assumed that the children are affected by the situation and the parent’s distress. However, many further questions about the children’s situation, as well as the parent’s, was assumed to be an extra burden on the families. Therefore, the children’s exposure to other adversities, such as their parents’ possible mental health problems, was not assessed, and could also be hypothesized to be another plausible explanation other than secondary traumatization to explain the findings in the current study. Future studies would benefit from a larger sample size. 

Thirdly, studies examining stalking survivors show that survivors who experience the most distress do not participate in studies as often as survivors who are less affected by the stalking [2]. With this in mind, we expect that parents may not enroll the most distressed children in a study because of the perceived risk of perpetuating the distress cycle. The study also did not distinguish between prior stalking and ongoing stalking, but only asked about the child’s symptoms within the last month. Also, it has not been possible to determine whether the high levels of PTSD in the children could also be the result of other traumatic experiences, such as possible family violence in the stalking situation. Finally, stalker typology data was not collected from participants, making it difficult to ascertain if stalker typology affects children differentially. Future research may benefit from investigating who the stalker is, and how this may influence the child. Since 95% of survivors of stalking were either married to, separated/divorced from, or an ex-partner of the perpetrator, one could hypothesize that this could also be another explanation or even possibly a primary contributing factor to the post-traumatic stress symptoms in children found in the current study. This should be further investigated, and we would anticipate that parent-stalking-parent may precede additional problems, as opposed to when the stalker is unrelated to the child.

In general, the study shows some limitations. There is a lack of direct evidence showing that PTSD symptoms of the sample is tied to parents being stalked; however, there is a lot of strong, indirect evidence. Even though further research is called for that may be able to address the current limitations, it is believed that the current pilot study provides sufficient and important information to the understanding of the impact of parents’ stalking on their children, which is something that research has overlooked until now.

## 6. Conclusions

The present pilot study found that children of stalking survivors demonstrated a high number of PTSD symptoms. Fifty-six percent of the overall sample met the criteria for PTSD. Of the three age groups investigated, schoolchildren reported the highest prevalence of PTSD symptoms when compared to their preschool and adolescent counterparts (85%, 22%, and 58% respectively). 

Arousal cluster symptoms were reported prevalently across the three participant groups, with the two older groups additionally reporting high concentration difficulties. The most reported symptoms across the three groups encompassed sleeping difficulties, intrusive thoughts and irritability. The mothers of the 0–6-year-olds reported that 61% of the preschool children were affected by stalking, and they perceived the observed symptoms as having an adverse effect on the child´s daily functioning. Half of the adolescents said that the symptoms they experienced created difficulties in their daily life. The present study is, to our knowledge, the first study to quantitatively investigate children of stalking survivors with standardized tests. Despite the small sample and the comparing of different age groups using different measures, these findings shed light on the fact that children could be significantly affected by parents being a stalking survivor, and that secondary stalking can create posttraumatic symptoms in the children. This can be explained by the theory of secondary traumatization. The limitations underline the need of further studies with larger sample sizes to examine the symptoms in children of stalking survivors. Furthermore, further study on this topic is important for exploring the best treatment for such children.

## Figures and Tables

**Table 1:** Descriptive statistics of Post-Traumatic Stress Disorder (PTSD) symptoms for the age groups.

	Preschoolers, N = 18	Schoolchildren, N = 20	Adolescents, N = 19
**Cluster B, Re-experience**			
Intrusive Thoughts	7 (39%)	20 (10%)	14 (74%)
Repeated Play	4 (22%)	11 (55%)	-
Trauma Specific Behavior	6 (33%)	-	-
Nightmares Trauma Related	2 (11%)	13 (65%)	-
Nightmares in General	6 (33%)	18 (90%)	8 (42%)
Flashbacks	6 (33%)	18 (90%)	12 (63%)
Dissociation	9 (50%)	-	-
Emotional Reactions	9 (50%)	16 (80%)	13 (68%)
Physiological Reactions	8 (44%)	16 (80%)	9 (47%)
**Cluster C, Avoidance**			
External Stimuli Avoidance	6 (33%)	15 (75%)	12 (63%)
Internal Stimuli Avoidance	6 (33%)	16 (80%)	12 (63%)
Memory Lapses	5 (28%)	15 (75%)	10 (53%)
Dislikes to Usual Likes	3 (17%)	13 (65%)	9 (47%)
Emotional Limitations	4 (22%)	16 (80%)	-
Feeling of a Limited Future	1 (6%)	15 (75%)	-
Social Withdrawal	7 (39%)	15 (75%)	9 (47%)
**Cluster D, Arousal**			
Trouble Falling Asleep	12 (67%)	-	-
Trouble Sleeping at Night	9 (50%)	19 (95%)	13 (68%)
Irritability	9 (50%)	20 (100%)	13 (68%)
Concentration	5 (28%)	18 (90%)	13 (68%)
Hypervigilance	9 (50%)	15 (75%)	13 (68%)
Exaggerated Startle	12 (67%)	13 (68%)	13 (68%)
Risk Behavior	-	-	4 (21%)
**Symptoms as a Problem**	11 (61%)	-	10 (53%)

## References

[1] Narud K., Friestad C., Dahl A. (2013). Stalking Experiences and Associated Factors – A Controlled Population-Based Study from Norway. Nordic J. Psychiatry.

[2] Jørgensen T. (2013). Omfanget og Karakteren af Stalking: En befolkningsundersøgelse.

[3] Schandorph S., Elklit A. (2013). Med barnet som gidsel—Stalking af Mødre.

[4] Justitsministeriet & Ministeriet for Børn, Undervisning og Ligestilling (2016). Stop Stalking—En styrket Indsat mod Stalking, Forfølgelse og Chikane.

[5] Dressing H., Kuehner K., Gass P. (2005). Lifetime Prevalence and impact of stalking in a European population. Br. J. Psychiatry.

[6] Johansen K., Tjørnhøj-Thomsen T., Helweg-Larsen K. (2013). Stalking i Danmark. En Kortlægning af Erfaringer, Konsekvenser og Støttebehov.

[7] Nikupeteri A., Laitinen M. (2015). Children’s Everyday Lives Shadowed by Stalking: Postseparation Stalking Narratives of Finnish Children and Women. Violence Vict..

[8] De Young A.C., Kenardy J.A., Cobham V.E. (2011). Trauma in Early Childhood: A Neglected Population. Clin. Child Fam. Psychol. Rev..

[9] Cohen J.A., Mannarino A.P., Deblinger E. (2006). Treating Trauma and Traumatic Grief in Children and Adolescents.

[10] Green J., McLaughlin K., Berglund P., Gruber M., Sampson N., Zaslavsky A., Kessler R. (2010). Childhood Adversities and Adult Psychopathology in the National Comorbidity Survey Replication (NCS-R) I: Associations with the First Onset of DSM-IV Disorders. Arch. Gener. Psychiatry.

[11] Kerig P.K., Fedorowicz A.E., Brown C.A., Warren M. (2000). Assessment and Intervention for PTSD in Children Exposed to Violence. J. Aggress. Maltreatment Trauma.

[12] Scott K., von Korff M., Angermeyer M., Benjet C., Bruffaerts R., de Girolamo G., Haro J., Lépine J., Ormel J., Posada-Villa J. (2011). Association of Childhood Adversities and Early-Onset Mental Disorders with Adult-Onset Chronic Physical Conditions. Arch. Gener. Psychiatry.

[13] Kellermann N.P.F. (2001). Transmission of Holocaust trauma—An integrative view. Israel J. Psychiatry.

[14] Schwerdtfeger K.L., Goff B.S.N. (2007). Intergenerational transmission of trauma: Exploring mother-infant prenatal attachment. J. Trauma. Stress.

[15] American Psychiatric Association (2013). Diagnostic and Statistical Manual of Mental Disorders.

[16] Johansen K., Tjørnhøj-Thomsen T. (2016). The Consequences of Coping with Stalking—Results from the First Qualitative study on Stalking in Denmark. Int. J. Public Health.

[17] Elklit A., Sørensen S.D. (2003). De Glemte Generationer—Perspektiver på tværgenerationel traumetransmission. Psyke Logos.

[18] Terr L.C. (1991). Childhood Traumas: An Outline and Overview. Am. J. Psychiatry.

[19] Herman J.L. (1992). Complex PTSD: A syndrome in survivors of prolonged and repeated trauma. J. Traum. Stress.

[20] Scheeringa M., Myers L., Putnam F., Zeanah C. (2012). Diagnosing PTSD in Early Childhood, an Empirical Assessment of Four Approaches. J. Trauma. Stress.

[21] American Psychiatric Association (2000). Diagnostic and Statistical Manual of Mental Disorders.

[22] Margolin G., Vickerman K.A. (2011). Posttraumatic Stress in Children and Adolescents Exposed to Family Violence: I. Overview and Issues. Couple Fam. Psychol. Res. Pract..

[23] Gaensbauer T. (1995). Trauma in the preverbal period. Symptoms, memoires and developmental impact. Psychoanal. Study Child.

[24] Pynoos R.S., Steinberg A.M., Goenjian A., van der Kolk B.A., McFarlane A.C., Weisaeth L. (2007). Traumatic Stress in Childhood and Adolescence: Recent Developments and Current Controversies. Traumatic Stress: The Effects of Overwhelming Experience on Mind, Body, and Society.

[25] Margolin G., Vickerman K.A. (2007). Post-traumatic Stress in Children and Adolescents Exposed to Family Violence: I. Overview and Issues. Prof. Psychol. Res. Pract..

[26] Pynoos R.S., Steinberg A.M., Layne C.M., Briggs E.C., Ostrowski S.A., Fairbank J.A. (2009). DSM-V PTSD Diagnostic Criteria for Children and Adolescents: A Developmental Perspective and Recommendations. J. Trauma. Stress.

[27] Scheeringa M., Haslett N. (2010). The Reliability and Criterion Validity of the Diagnostic Infant and Preschool Assessment: New Diagnostic Instrument for Young People. Child Psychiatry Hum. Dev..

[28] Løkkegaard S., Andersen S., Elklit A. Is Trauma and Post-Traumatic Stress Disorder Connected to Psychiatric Comorbidity in Danish Preschoolers?.

[29] Løkkegaard S., Elmose M., Elklit A. Validation of the Diagnostic Infant and Preschool Assessment in a Danish, Trauma-Exposed Sample of Young Children.

[30] Geller P., Neugebauer R., Possemato A., Walter P., Dummit E., Silva R. (2007). Psychometric Properties of Darryl, a Cartoon Based Measure to Assess Community Violence Related PTSD in Children. Psychiatr. Q..

[31] Løkkegaard S., Rønholt S., Karsberg S., Elklit A. (2016). Validation of the PTSD Screening Cartoon Test “Darryl” in a Danish Clinical Sample of Children and Adolescents. Int. J. Methods Psychiatr. Res..

[32] Elklit A., Fuglsang A. (2001). En Oversigt over Dansk Psykotraumatologi.

[33] Mollica R., Caspi-Yavin Y., Bollini P., Truong T., Tor S., Lavelle J. (1992). The Harvard Trauma Questionnaire. Validation a Cross-Cultural Instrument for Measuring Torture, Trauma and Posttraumatic Stress Disorder in Indochinese Refugees. J. Nervous Ment. Dis..

[34] Elklit A., O’Connor M. (2005). Post-traumatic stress disorder in a Danish population of elderly bereaved. Scand. J. Psychol..

[35] Elklit A., Brink O. (2003). Acute Stress Disorder in Physical Assault Victims Visiting a Danish Emergency Ward. Violence Vict..

[36] Bach M.E. (2003). En empirisk undersøgelse og analyse af ”Emotional numbing” som eventuel selvstænding faktor i PTSD. Psykologisk Studieskriftserie.

[37] Kitzmann K.M., Gaylord N.K., Holt A.R., Kenny E.D. (2003). Child Witnesses to Domestic Violence: A Meta-Analytic Review. J. Consult. Clin. Psychol..

[38] Duch C., Elklit A. (2008). Børneperspektiv på en katastrofe. Psykolognyt.

[39] Scheeringa M., Zeanah C., Myers L., Putnam F. (2003). New Findings on Alternative Criteria for PTSD in Preschool Children. J. Am. Acad. Child Adolesc. Psychiatry.

[40] Wolf N.M., Løkkegaard S.S., Elklit A. (2018). Psychological Distress and Attachment Insecurity of Stalked Mothers. J. Interpersonal Violence.

